# Supplementary material for: Association between Depressive Symptoms and Food Insecurity among Indonesian Adults: Results from the 2007–2014 Indonesia Family Life Survey
Source: Nutrients. 2019 Dec 11;11(12):3026. doi: 10.3390/nu11123026 (PMC6950164; doi:10.3390/nu11123026)
Supplement: Supplementary file 1 [file nutrients-11-03026-s001.pdf]

nutrients MDPI

Tabel S 1. One-way ANOVA with Bonferroni Post-Hoc Test Results year 2014.

Variables	df		F	2014					
				Acceptable X Borderline		Acceptable X Poor		Borderline X Poor	
	BG	WG		M	p	M	p	M	p
Body Mass Index (kg/m^2)	2	8610	25.12	0.68	< 0.001	0.89	< 0.001	0.21	0.139
Body Shape Index (m$^{11/6}$ kg$^{-2/3}$)	2	6577	0.32	9.30 x 10-5	1.000	1.47 x 10-4	1.000	5.50 x 10-5	1.000
Waist Circumference (cm)	2	6577	23.38	1.77	< 0.001	2.59	< 0.001	0.82	0.034
Systolic BP (mmHg)	2	8610	7.21	-1.77	0.040	-2.57	< 0.001	-0.81	0.477
Diastolic BP (mmHg)	2	8610	0.76	-0.13	1.000	-0.42	0.809	-0.29	1.000
Food Consumption Score	2	8610	11115.74	16.12	< 0.001	33.29	< 0.001	17.18	< 0.001
Walking PA Days	2	8610	1.91	0.03	1.000	0.14	0.297	0.12	0.354
Moderate PA Days	2	8610	6.74	0.06	1.000	0.26	0.005	0.20	0.011
Vigorous PA Days	2	8610	3.99	-0.18	0.016	-0.09	0.414	0.09	0.257
CES-D 10 Score	2	8610	16.60	0.34	0.075	0.78	< 0.001	0.44	0.001

Abbreviation: BG, between group; WG, within group; M, mean different between group; p, p-value between group; CES-D 10, Center for Epidemiological Studies Depression 10 items; SD, standard deviation.

nutrients

In 2014, the mean body mass index (BMI) was significantly different between the food consumption groups ($F (2, 8610) = 25.12$, $p < 0.001$). Post hoc comparisons using the Bonferroni test were carried out. BMI in the acceptable group was significantly different from the BMI in the borderline group. BMI in the acceptable group was also significantly different from the BMI in the poor group ($p < 0.001$). However, the BMI of the borderline group did not significantly differ from the BMI of the poor group. The mean waist circumference (WSC) was significantly different between the food consumption groups ($F (2, 4658) = 23.38$, $p < 0.001$). The WSC in the acceptable group was significantly different from the WSC in the borderline or the poor group ($p = 0.034$ - < 0.001).

The mean systolic blood pressure (SBP) was significantly different between the food consumption groups ($F (2, 8610) = 7.21$, $p < 0.001$). The SBP in the acceptable group was significantly different from the SBP in the borderline group ($p = 0.04$). The SBP in the acceptable group was also significantly different from the SBP in the poor group ($p < 0.001$). However, the SBP of the borderline group did not significantly differ from the SBP of the poor group. The mean food consumption score (FCS) was significantly difference between the food consumption groups ($F (2, 8610) = 11{,}115.74$, $p < 0.001$). The FCS in the acceptable group was significantly different from the FCS in the borderline or the poor group ($p < 0.001$).

The mean moderate physical activity (MPA) days was significantly different between the food consumption groups ($F (2, 8610) = 6.74$, $p = 0.001$). The MPA days in the borderline group were significantly different from the MPA days in the poor group ($p = 0.011$). The MPA days in the acceptable group was also significantly different from the MPA days in the poor group ($p = 0.005$). However, the MPA days of the borderline group did not significantly differ from the MPA days of the acceptable group. On the other hand, the mean vigorous physical activity (VPA) days was significantly different between the food consumption groups ($F (2, 8610) = 3.99$, $p = 0.019$). The VPA days in the acceptable group were only significantly different from the VPA in the borderline group ($p = 0.016$) but not in the other groups.

The mean CES-D score was significantly different between the food consumption groups ($F (2, 8610) = 16.60$, $p < 0.001$). The CES-D score in the borderline group was significantly different from the CES-D score in the poor group ($p = 0.001$). The CES-D score in the acceptable group was also significantly different from the CES-D score in the poor group ($p < 0.001$). However, the CES-D score of the acceptable group did not significantly differ from the CES-D score of the borderline group.

nutrients MDPI

Tabel S 2. One-way ANOVA with Bonferroni Post-Hoc Test Results year 2007.

Variables	df		F	2007					
				Acceptable X Borderline		Acceptable X Poor		Borderline X Poor	
	BG	WG		M	p	M	p	M	p
Body Mass Index (kg/m^2)	2	8610	16.93	0.35	0.189	0.81	< 0.001	0.45	< 0.001
Body Shape Index (m$^{11/6}$ kg$^{-2/3}$)	2	4658	3.58	9.97 x 10-4	0.037	8.62 x 10-4	0.035	-1.35 x 10-4	1.000
Waist Circumference (cm)	2	4658	18.83	1.59	0.051	3.12	< 0.001	1.52	0.001
Systolic BP (mmHg)	2	8610	1.69	-0.42	1.000	-1.14	0.419	-0.71	0.596
Diastolic BP (mmHg)	2	8610	0.11	0.24	1.000	0.16	1.000	-0.08	1.000
Food Consumption Score	2	8610	4313.01	14.25	< 0.001	45.64	< 0.001	31.39	< 0.001
Walking PA Days	2	8610	1.33	0.08	1.000	-0.04	1.000	-0.12	0.311
Moderate PA Days	2	8610	6.60	0.44	0.001	0.35	0.004	-0.09	0.664
Vigorous PA Days	2	8610	1.57	0.23	0.229	0.15	0.530	-0.08	1.000
CES-D 10 Score	2	8610	20.95	0.29	0.177	0.70	< 0.001	0.41	< 0.001

Abbreviation: BG, between group; WG, within group; M, mean different between group; p, p-value between group; CES-D 10, Center for Epidemiological Studies Depression 10 items; SD, standard deviation.

nutrients

In 2007, the mean BMI was significantly different between the food consumption groups (F (2, 8610) = 16.93, $p < 0.001$). The BMI in the acceptable group was significantly different from the BMI in the poor group. The BMI in the borderline group was also significantly different from the BMI in the poor group ($p < 0.001$). However, the BMI of the acceptable group did not significantly differ from the BMI of the borderline group. The mean body shape index (BSI) was significantly different between the food consumption groups (F (2, 8610) = 3.58, $p < 0.001$). BSI in the acceptable group was significantly different from the BSI in the borderline group ($p = 0.037$). BSI in the acceptable group was also significantly different from the BSI in the poor group ($p = 0.035$). However, the BSI of the borderline group did not significantly differ from the BSI of the poor group. The mean WSC was significantly different between the food consumption groups (F (2, 4658) = 18.83, $p < 0.001$). The WSC in the acceptable group was significantly different from the WSC in the poor group ($p < 0.001$). WSC in the borderline group was also significantly different from the WSC in the poor group ($p = 0.001$). However, the WSC of the acceptable group did not significantly differ from the WSC of the borderline group.

The mean FCS was significantly difference between the food consumption groups (F (2, 8610) = 4313.01, $p < 0.001$). The FCS in the acceptable group was significantly different from the FCS in the borderline or the poor group ($p < 0.001$). The mean MPA days was significantly different between the food consumption groups (F (2, 8610) = 6.60, $p = 0.001$). The MPA days in the acceptable group were significantly different from the MPA days in the borderline group ($p = 0.001$). The MPA days in the acceptable group was also significantly different from the MPA days in the poor group ($p = 0.004$). However, the MPA days of the borderline group did not significantly differ from the MPA days of the poor group.

Furthermore, the mean CES-D score was significantly different between the food consumption groups (F (2, 8610) = 20.95, $p < 0.001$). The CES-D score in the borderline group was significantly different from the CES-D score in the poor group ($p < 0.001$). The CES-D score in the acceptable group was also significantly different from the CES-D score in the poor group ($p < 0.001$). However, the CES-D score of the acceptable group did not significantly differ from the CES-D score of the borderline group.

© by the authors. Licensee MDPI, Basel, Switzerland. This article is an open access article distributed under the terms and conditions of the Creative Commons Attribution (CC BY) license (http://creativecommons.org/licenses/by/4.0/).
